# From Natural Product to Topical Antimicrobial Candidate: Evaluating *Nigella sativa* Seed Oil as a Broad-Spectrum Topical Antimicrobial in Multi-Tiered Preclinical Models

**DOI:** 10.3390/ph19070986

**Published:** 2026-06-25

**Authors:** Faris S. Alnezary, Masaad Saeed Almutairi

**Affiliations:** 1 Department of Pharmacy Practice, College of Pharmacy, Taibah University, Madinah 41477, Saudi Arabia; 2 Department of Pharmacy Practice, College of Pharmacy, Qassim University, Qassim 51452, Saudi Arabia

**Keywords:** *Nigella sativa*, thymoquinone, MRSA, *Pseudomonas aeruginosa*, polymicrobial biofilms, preclinical models, infectious disease

## Abstract

**Background:** Polymicrobial skin and soft tissue infections (SSTIs) are frequently complicated by methicillin-resistant *Staphylococcus aureus* (MRSA) and co-colonizing Gram-negative pathogens like *Pseudomonas aeruginosa* (*P. aeruginosa*). Mupirocin, the clinical gold standard, is limited by rising resistance and an intrinsic “mupirocin gap” against *P. aeruginosa*. This study evaluates a novel *Nigella sativa* (NS) seed oil topical formulation as an alternative. **Methods:** A 4-tier preclinical platform assessed the NS formulation against MRSA, methicillin-sensitive *S. aureus* (MSSA), *Streptococcus pyogenes*, and *P. aeruginosa*. The pipeline included: (1) in vitro agar diffusion, (2) a gauze biofilm prevention model, (3) an ex vivo porcine ear skin model challenging epidermal lipid barriers, and (4) an in vivo *Galleria mellonella* model evaluating trans-cuticular systemic protection. **Results:** The NS formulation produced extensive diffusion zones, completely inhibiting *S. pyogenes* and outperforming controls against MSSA and *P. aeruginosa*. In the gauze model, NS achieved complete eradication of MSSA and *S. pyogenes*, while significantly suppressing MRSA and *P. aeruginosa* biofilms (*p* < 0.001). In the ex vivo porcine model, NS yielded >1.5 to >2.5 log reductions across all pathogens at 24 h (*p* < 0.001). Furthermore, in the in vivo *G. mellonella* model, topical NS significantly reduced the systemic bioburden of MSSA, *S. pyogenes*, and *P. aeruginosa* (*p* < 0.001), though MRSA reduction lacked statistical significance. **Conclusions:** The novel NS formulation demonstrates potent broad-spectrum antimicrobial activity. By effectively bridging the “mupirocin gap” against *P. aeruginosa* and demonstrating significant efficacy against MRSA in in vitro and ex vivo environments, it represents a promising plant-based pre-clinical candidate that strongly warrants future evaluation in live mammalian wound healing models.

## 1. Introduction

The escalating incidence of community-onset and healthcare-associated skin and soft tissue infections (SSTIs) poses a formidable and growing threat to global public health. *Staphylococcus aureus* (*S. aureus*) is the predominant bacterial etiology of purulent SSTIs, responsible for up to 76% of such infections, ranging from minor superficial lesions to severe, limb- and life-threatening conditions including complicated abscesses and necrotizing fasciitis [1,2]. The clinical management of these infections has been severely compromised by the widespread dissemination of methicillin-resistant *S. aureus* (MRSA), which frequently exhibits multidrug resistance to standard systemic therapies, including macrolides and tetracyclines [3]. Beyond monomicrobial staphylococcal infections, polymicrobial wound environments—particularly thermal burn injuries and diabetic ulcers—present a compounded therapeutic challenge. In these complex wounds, MRSA frequently co-colonizes with virulent Gram-negative pathogens, most notably *Pseudomonas aeruginosa* (*P. aeruginosa*), leading to delayed wound healing, graft failure, and an increased risk of progression to systemic bacteremia and sepsis [4]. The ability of these pathogens to establish persistent colonization in the anterior nares and on the skin serves as a primary reservoir for both autoinfection and nosocomial transmission [2,3].

To mitigate the risk of recurrent SSTIs and prevent transmission, topical antimicrobial decolonization strategies have been widely implemented, with mupirocin serving as the clinical “gold standard” for the eradication of *S. aureus* [1,2]. However, the extensive prophylactic and therapeutic use of mupirocin has exerted profound selective pressure, driving a rapid and concerning emergence of mupirocin resistance. High-level mupirocin resistance, mediated by the plasmid-borne *mupA* gene which encodes a novel isoleucyl-tRNA synthetase, directly correlates with clinical decolonization failure and persistent MRSA carriage [1,2,5]. In pediatric populations with recurrent SSTIs, mupirocin resistance rates have been reported as high as 14.7% to 18% [1,2]. Furthermore, this plasmid-mediated resistance is frequently co-harbored with other resistance determinants, such as those for clindamycin, signaling a shift toward highly resilient, multidrug-resistant lineages [1,5]. Crucially, the utility of mupirocin is further limited by the “mupirocin gap”: an intrinsic lack of efficacy against Gram-negative pathogens such as *P. aeruginosa*. While combinatorial topical agents, such as triple antibiotic ointments containing neomycin, attempt to bridge this gap, their widespread use is increasingly discouraged due to high rates of allergic contact dermatitis and variable efficacy against resistant staphylococci [6]. Consequently, there is an urgent, unmet clinical need for novel, broad-spectrum topical antimicrobials capable of overcoming high-level mupirocin resistance and eradicating polymicrobial wound flora.

Given the therapeutic failures associated with conventional single-target antibiotics and the “mupirocin gap,” the exploration of multi-target, plant-based therapeutics has gained considerable traction. NS, and specifically its primary bioactive quinone constituent, thymoquinone (TQ), has emerged as a promising broad-spectrum antimicrobial compound with the capability of overcoming multidrug-resistant pathogens [7,8,9]. Thymoquinone exhibits exceptional bactericidal activity against MRSA by inducing structural damage to the bacterial cell wall and membrane, while demonstrating a markedly low propensity for selecting resistant mutants, unlike standard antibiotics [10]. Crucially, TQ directly combats resistance mechanisms by acting as an efflux pump inhibitor, significantly impairing the activity of the *norA* efflux pump in multidrug-resistant *S. aureus* and down-regulating the *mepA* efflux gene, thereby restoring susceptibility to conventional antibiotics [9,11]. Furthermore, TQ bridges the therapeutic gap by exhibiting potent anti-virulence and anti-biofilm properties against Gram-negative *P. aeruginosa*; it disrupts mature biofilms, inhibits swarming motility, and significantly attenuates the production of tissue-damaging virulence factors (e.g., pyocyanin, proteases, and elastase) by down-regulating critical quorum sensing genes (*lasI*, *lasR*, *rhlI*, and *rhlR*) [7,12]. The demonstrated activity of NS oil and TQ in eradicating established biofilms, penetrating extracellular polymeric matrices, and enhancing local antioxidant defenses suggest that they may represent promising candidates for advanced topical wound dressings and decolonization strategies [13,14].

The baseline antibacterial, antibiofilm, and anti-inflammatory properties of NS seed oil and its primary bioactive constituent, thymoquinone, have been extensively documented in recent literature [15,16,17,18]. However, the development and translation of these promising botanical bioactives into clinical practice are frequently hindered by a reliance on overly simplistic in vitro planktonic assays, such as standard minimal inhibitory concentration (MIC) testing, which fail to accurately predict in vivo efficacy. True chronic wound environments are defined by complex biological and physical barriers—including tissue exudates, host skin lipids, the stratum corneum, and mature, structured biofilms—that can sequester, deactivate, or impede the penetration of topical drugs [19]. Furthermore, bacteria encased within an extracellular polysaccharide matrix in mature biofilms display extreme tolerance to antimicrobial therapies, often surviving antibiotic concentrations 500 to 5000 times higher than those required to kill their planktonic counterparts [20]. To bridge this translational gap, researchers are increasingly utilizing advanced ex vivo and in vivo preclinical models. Ex vivo porcine skin explants are highly homologous to human skin and provide an ideal biological substrate and nutritional source to mimic the lipid barriers and robust extracellular matrix architecture of mature wound biofilms [19,20]. Furthermore, the in vivo *Galleria mellonella* (greater wax moth) larvae model has emerged as a valuable, ethical, and high-throughput alternative to mammalian testing, allowing for the comprehensive assessment of systemic protection, toxicity, and trans-cuticular antimicrobial efficacy without the strict ethical constraints associated with vertebrate models [20].

Therefore, the scientific novelty and primary objective of the present study lie in the translational pre-clinical evaluation of this uniquely optimized 5% (*w*/*w*) NS hydrogel formulation. Unlike conventional complex nanoformulations, this preparation achieves the absolute maximum stable loading capacity of the lipophilic active within a standardized, water-soluble hydrogel matrix without requiring synthetic chemical emulsifiers, thereby preserving a non-irritating phytocomplex. By utilizing a rigorous, multi-tiered screening platform—progressing from in vitro agar diffusion and gauze biofilm prevention to highly translational ex vivo porcine skin explants and an in vivo *Galleria mellonella* systemic infection model—this study aims to establish a robust pre-clinical proof-of-concept. Crucially, this study seeks to demonstrate the formulation’s novel capacity to effectively bridge the critical ‘mupirocin gap’ by eradicating resilient Gram-positive pathogens while simultaneously exhibiting demonstrating activity against intrinsically resistant *P. aeruginosa* within complex, tissue-like environments.

## 2. Results

### 2.1. Agar Well Diffusion

**Figure 1:**
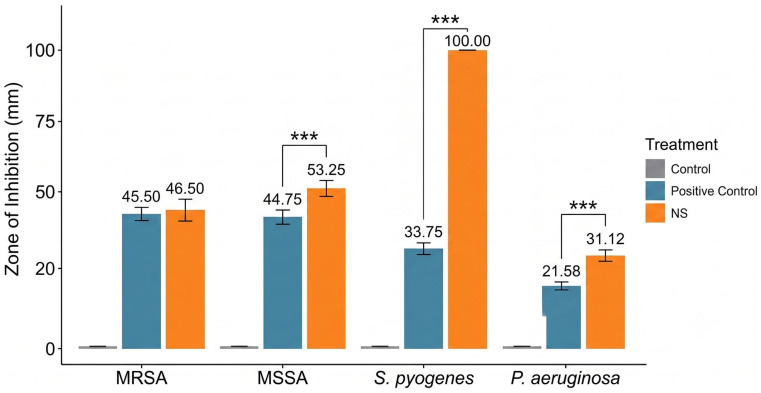
In vitro antimicrobial activity via agar well diffusion. Clearance zones were measured following 24 h of exposure. Bars represent the mean clearance zone diameter (mm), error bars indicate the standard deviation (SD), and individual data points are superimposed to illustrate experimental variability across four independent biological replicates (*n* = 4). Asterisks denote statistical significance (*** *p* < 0.001).

The novel NS seed oil formulation demonstrated potent, broad-spectrum release characteristics in the in vitro diffusion assay. Against MRSA, the NS formulation produced a mean zone of inhibition of 46.50 ± 0.62 mm, comparable to the positive control mupirocin (45.50 ± 0.34 mm; *p* = 0.171, 95% CI [−2.31, 4.31], Hedges’ g = 0.45). The NS formulation demonstrated significantly superior efficacy against methicillin-sensitive *S. aureus* (MSSA) with a mean zone of 53.25 ± 0.72 mm compared to 44.75 ± 0.45 mm for mupirocin (*p* < 0.001, 95% CI [4.54, 12.46], Hedges’ g = 3.23). Notably, the experimental agent achieved complete plate clearance (recorded as 100.00 ± 0.00 mm to reflect the entire dish diameter) against *Streptococcus pyogenes*, significantly outperforming the positive control (33.75 ± 1.17 mm; *p* < 0.001, 95% CI [60.74, 71.76], Hedges’ g = 18.10). Furthermore, NS exhibited robust activity against the Gram-negative pathogen *P. aeruginosa*, producing a mean zone of 31.12 ± 0.34 mm, which significantly outperformed the triple antibiotic control (21.58 ± 0.31 mm; *p* < 0.001, 95% CI [7.39, 11.71], Hedges’ g = 6.65) (Figure 1).

### 2.2. Gauze Model (Biofilm Prevention)

**Figure 2:**
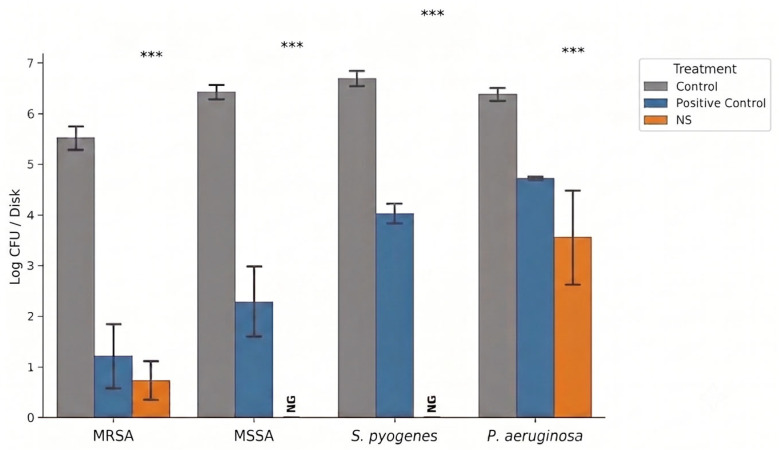
Prevention of nascent bacterial biofilm formation in the in vitro gauze model. Orange bars represent the experimental NS formulation. “NG” indicates No Growth, representing complete inhibition falling below the assay’s limit of detection (10 CFU/disk). Bars represent the mean log CFU/disk, error bars indicate the standard deviation (SD), and individual data points are superimposed across four independent biological replicates (*n* = 4). Asterisks denote statistical significance (*** *p* < 0.001).

In the gauze contact assay, used to evaluate penetration and biofilm prevention through standard wound dressings, vehicle control disks developed robust biofilms across all tested strains. Treatment with the NS formulation resulted in complete bacterial eradication (0 CFU/disk) for both MSSA and *S. pyogenes*, demonstrating highly significant log reductions compared to controls (MSSA: *p* < 0.001, 95% CI [−7.08, −5.76], Hedges’ g = −14.56; *S. pyogenes*: *p* < 0.001, 95% CI [−7.40, −5.98], Hedges’ g = −14.14). The NS formulation also profoundly suppressed MRSA biofilm formation, reducing the bacterial burden to a mean of 200 CFU (0.58 ± 0.39 Log CFU) compared to controls (5.57 ± 0.46 Log CFU; *p* < 0.001, 95% CI [−6.87, −2.71], Hedges’ g = −3.47). Against *P. aeruginosa*, the NS formulation maintained potent antibiofilm activity (3.51 ± 0.56 Log CFU), representing a highly significant reduction compared to vehicle controls (6.33 ± 0.22 Log CFU; *p* < 0.001, 95% CI [−7.38, −0.79], Hedges’ g = −1.86) (Figure 2).

### 2.3. Ex Vivo Pig Ear Model

**Figure 3:**
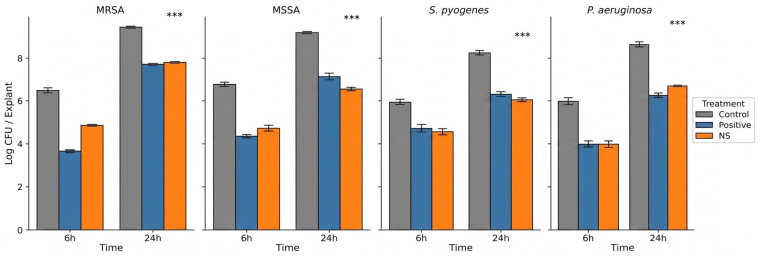
Evaluation of the 5% NS formulation in the ex vivo porcine skin explant model. Bioburden reduction was quantified following topical application to assess efficacy against acute tissue colonization. Bars represent the mean log CFU/explant recovery, error bars indicate the standard deviation (SD), and individual data points are superimposed across four independent biological replicates (*n* = 4). Asterisks denote statistical significance (*** *p* < 0.001).

The ex vivo porcine skin model validated the formulation’s capacity to maintain potent bioactivity in the presence of complex skin lipids over a 24 h period. For MRSA, the NS treatment significantly reduced the bioburden from 9.49 ± 0.02 Log CFU in the vehicle control to 7.82 ± 0.04 Log CFU, achieving a 1.67-log reduction (*p* < 0.001, 95% CI [1.46, 1.87], Hedges’ g = 12.29). The formulation demonstrated an antimicrobial activity against MSSA, yielding a 2.65-log reduction (9.25 ± 0.05 to 6.60 ± 0.08 Log CFU; *p* < 0.001, 95% CI [2.25, 3.11], Hedges’ g = 9.45). Significant suppression was also observed against *S. pyogenes*, with a 2.22-log reduction (8.33 ± 0.08 to 6.11 ± 0.06 Log CFU; *p* < 0.001, 95% CI [1.68, 2.74], Hedges’ g = 6.27). Importantly, the NS formulation maintained broad-spectrum efficacy against the Gram-negative pathogen *P. aeruginosa*, achieving a 2.06-log reduction compared to vehicle controls (8.76 ± 0.05 to 6.70 ± 0.02 Log CFU; *p* < 0.001, 95% CI [1.46, 2.47], Hedges’ g = 5.85) (Figure 3).

### 2.4. Galleria mellonella Topical Treatment

**Figure 4:**
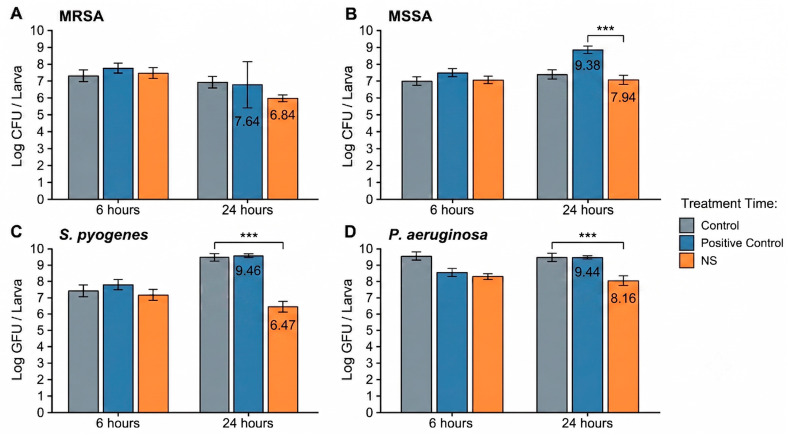
Assessment of systemic bacterial burden in the in vivo Galleria mellonella model. Bioburden was quantified from extracted hemolymph 24 h post-infection and topical application to evaluate trans-cuticular efficacy against: (**A**) Methicillin-resistant Staphylococcus aureus (MRSA); (**B**) Methicillin-sensitive *S. aureus* (MSSA); (**C**) Streptococcus pyogenes; and (**D**) Pseudomonas aeruginosa. Bars represent the mean log CFU/larva, error bars indicate the standard deviation (SD), and individual data points are superimposed to illustrate experimental variability across four independent biological replicates (*n* = 4). Asterisks denote statistical significance (*** *p* < 0.001), while “NS” indicates non-significance (*p* ≥ 0.05).

Topical application of the NS formulation in the *Galleria mellonella* in vivo model evaluated trans-cuticular protective efficacy and systemic infection mitigation at 24 h. The formulation significantly reduced the systemic burden of *S. pyogenes*, reducing counts from 9.46 ± 0.23 Log CFU down to 6.47 ± 0.04 Log CFU, representing a highly significant 2.99-log reduction (*p* < 0.001, 95% CI [2.45, 3.53], Hedges’ g = 13.91). Against MSSA, the systemic load was effectively reduced by 1.44 logs (9.38 ± 0.05 to 7.94 ± 0.32 Log CFU; *p* < 0.001, 95% CI [1.02, 1.86], Hedges’ g = 5.75). Against *P. aeruginosa*, topical application successfully reduced internal counts by 1.28 logs compared to vehicle controls (9.44 ± 0.13 to 8.16 ± 0.05 Log CFU; *p* < 0.001, 95% CI [0.98, 1.58], Hedges’ g = 8.52). While the NS formulation reduced the systemic MRSA burden by 0.80 logs (7.64 ± 0.44 to 6.84 ± 0.16 Log CFU), this specific reduction did not reach statistical significance (*p* = 0.111, 95% CI [−0.25, 1.85], Hedges’ g = 2.05) due to variability in the vehicle control group (Figure 4).

## 3. Discussion

This study establishes the efficacy of a novel 5% NS seed oil topical gel, representing a promising broad-spectrum pre-clinical candidate with the potential to serve as an adjunctive or alternative agent to standard therapies for the management of complex skin and soft tissue infections. By utilizing a rigorous, multi-tiered preclinical screening platform, we demonstrated that the NS formulation matched the bactericidal activity of the clinical gold standard, mupirocin, against Gram-positive pathogens (MRSA, MSSA, and *S. pyogenes*) while vastly outperforming it by achieving complete agar plate clearance against *S. pyogenes*. It is important to note that the exceptionally large diffusion profiles observed for the NS formulation are heavily facilitated by its water-soluble hydrogel base, which permits unhindered radial diffusion through the agar matrix, unlike the highly hydrophobic petrolatum bases of the standard mupirocin and triple antibiotic comparators [21,22]. Most importantly, the NS gel effectively bridged the therapeutic “mupirocin gap” by demonstrating an antimicrobial activity against *P. aeruginosa*, a pathogen intrinsically resistant to mupirocin. These findings, validated across in vitro diffusion, structural gauze biofilm, ex vivo porcine tissue, and in vivo invertebrate models, confirm that plant-based formulations utilizing NS seed oil can significantly suppress resilient Gram-positive and Gram-negative pathogens within complex, wound-like environments.

The management of polymicrobial wounds is heavily complicated by the “mupirocin gap.” While mupirocin has long been the primary topical agent for MRSA decolonization, its narrow spectrum leaves polymicrobial wounds vulnerable to Gram-negative colonization by pathogens such as *P. aeruginosa*, leading to chronic inflammation and delayed healing [6]. Furthermore, the escalating clinical threat of high-level, plasmid-mediated mupirocin resistance threatens the utility of this agent even against staphylococci, necessitating combinatorial approaches or entirely novel therapeutic alternatives [23]. Our findings in the gauze biofilm and porcine skin models highlight the critical advantage of the NS formulation in overcoming these limitations. While mupirocin failed to achieve complete prevention in the gauze biofilm model and was wholly inactive against *P. aeruginosa*, the NS gel successfully penetrated standard cotton dressings and maintained potent bioactivity in the presence of skin lipids, achieving highly significant > 2-log to >4-log reductions across all tested strains. This demonstrates that the NS formulation successfully addresses both the resistance mechanisms hindering mupirocin and its intrinsic lack of Gram-negative coverage.

The robust efficacy of the 5% NS gel observed in this study is likely driven by the unique pharmacodynamics of delivering ultra-high local concentrations of plant-based bioactives. Based on established literature, the profound phenotypic efficacy observed in our models is likely mediated by the previously documented molecular properties of NS and its primary quinone constituent, thymoquinone, which have been widely reported to modify intrinsic resistance and act as potent efflux pump inhibitors [7,9]. Given the ultra-high local concentrations delivered by the 5% topical gel, it is highly probable that this targeted biochemical suppression acts synergistically with the non-specific membrane destabilization driven by the lipophilic bulk of the oil. Beyond this antimicrobial synergy, NS oil and its active component, thymoquinone, are documented to actively accelerate wound healing in burn and excisional models. By simultaneously reducing oxidative stress and modulating prolonged inflammation, the formulation provides a dual-action effect that transcends simple pathogen clearance, promoting tissue regeneration alongside infection control [13,24]. While systemic administration of antimicrobials often fails to reach therapeutic concentrations within the avascular, necrotic microenvironment of a chronic wound, topical hydrogels can deliver massive local drug gradients directly to the wound bed [25]. At these ultra-high local concentrations, the active constituents of NS can effectively overwhelm intrinsic bacterial resistance mechanisms and efflux pumps, achieving rapid bactericidal and anti-biofilm activity that systemic dosing cannot safely reach [26]. The ability of NS to formulate effectively into nanoemulgels has also been previously shown to improve targeted transdermal delivery and enhance diabetic wound closure [27]. While previous studies have highlighted the efficacy of isolated thymoquinone preparations [10,14] or complex NS nanoemulgels [27], these approaches often face challenges regarding manufacturing scalability and the rapid oxidation of purified constituents. The present study demonstrates that a straightforward, 5% (*w*/*w*) incorporation of cold-pressed NS oil into an inert, water-soluble hydrogel provides optimal diffusion properties without the need for sophisticated nanoparticle engineering. Unlike earlier preparations primarily tested against planktonic cells [10], this specific hydrogel vehicle was uniquely validated for its capacity to deliver ultra-high local concentrations capable of penetrating standard cotton wound dressings and overcoming epidermal lipid barriers to strictly prevent the formation of resilient polymicrobial biofilms.

The translational impact of these findings is heavily supported by the fidelity of the 4-tier preclinical pipeline utilized in this study. The development of topical antimicrobials is frequently hindered by a reliance on overly simplistic in vitro planktonic assays that fail to account for the physical and biological barriers of a true wound, such as the sequestering effects of wound debris, skin lipids, and protein-induced inactivation that often compromises the efficacy of topical agents [19]. By incorporating an ex vivo porcine skin model, which provides an accurate structural and lipid environment for mature MRSA and *P. aeruginosa* biofilms, we bypassed the limitations of abiotic surface testing [20]. Coupling this with the in vivo *Galleria mellonella* model provided critical validation of trans-cuticular systemic protection and in vivo survival. We propose that this trans-cuticular efficacy is driven by thermodynamic partitioning; the highly lipophilic bioactives of the NS oil rapidly partition out of the hydrophilic hydrogel vehicle and preferentially diffuse into the lipid-rich epicuticle of the larvae to reach the systemic hemolymph. Recent literature firmly establishes *G. mellonella* as a robust antimicrobial screening model that accurately reflects the in vivo toxicity and efficacy of novel agents [28,29]. Furthermore, *G. mellonella* has been shown to effectively recapitulate the hallmarks of burn trauma and localized infection seen in complex mammalian models [30]. Importantly, the use of slaughterhouse-derived porcine explants and invertebrate models creates a highly predictive preclinical pipeline that strictly adheres to the “3Rs” of animal research (Replacement, Reduction, Refinement), ethically bridging the gap between in vitro discovery and mammalian trials.

Despite these robust findings, this study is subject to several limitations that warrant further investigation. First, the translational utility of our models requires refinement: the *G. mellonella* model, while useful for screening, lacks an adaptive immune system and long-term longitudinal data, while our ex vivo porcine explants lack systemic circulation and inflammatory recruitment. Future studies should therefore utilize live mammalian models to evaluate dynamic healing markers, such as collagen deposition and epithelialization. Second, regarding microbiological scope, our foundational assays utilized mono-species inoculums and focused on biofilm prevention rather than the disruption of mature (48–72 h) extracellular matrices. Subsequent research must address these gaps by evaluating the formulation against polymicrobial biofilms and established matrices to better mirror the clinical environment of chronic wounds.

Third, pharmaceutical and safety profiling remain essential next steps. While our initial data confirms macroscopic efficacy, we have yet to perform formal cytotoxicity testing against human dermal fibroblasts and keratinocytes, nor have we conducted localized sensitization trials. Furthermore, to ensure clinical reproducibility and potency, future development must transition from a proof-of-concept vehicle to a standardized pharmaceutical formulation—incorporating rigorous pH, rheological, and release-kinetic characterization, alongside chemical standardization of key bioactives like thymoquinone. Finally, while we have established baseline phenotypic activity, mechanistic elucidation through transcriptomic profiling (e.g., RT-qPCR), and comparative efficacy trials against a broader panel of standard-of-care agents (e.g., silver sulfadiazine, polyhexanide), will be necessary to define the formulation’s precise therapeutic advantage in the clinical landscape.

## 4. Materials and Methods

### 4.1. Bacterial Strains and Antimicrobial Agents

To ensure a comprehensive evaluation of broad-spectrum antimicrobial efficacy, this study utilized a diverse panel of highly virulent skin and soft tissue pathogens. The test panel included MRSA (ATCC BAA-41; American Type Culture Collection, Manassas, VA, USA), a clinical isolate of MSSA, *Streptococcus pyogenes* (ATCC 19615), and the Gram-negative pathogen *P. aeruginosa* (ATCC 27853). All bacterial strains were maintained on tryptic soy agar (Thermo Fisher Scientific, Waltham, MA, USA) at 4 °C and routinely sub-cultured in Luria-Bertani (LB) broth (Sigma-Aldrich, St. Louis, MO, USA) to reach the logarithmic growth phase prior to experimental use. The primary experimental test agent was a novel 5% (*w*/*w*) NS seed oil topical formulation. Commercial mupirocin 2% ointment (GSK, Brentford, UK) served as the positive control for Gram-positive organisms (staphylococci and *S. pyogenes*), while triple antibiotic ointment (neomycin 3.5 mg/g, polymyxin B 5000 units/g, bacitracin 400 units/g) (Johnson & Johnson, New Brunswick, NJ, USA) served as the positive control for *P. aeruginosa*. A drug-free gel formulation (pure hydrogel matrix without active oil) served as the negative vehicle control across all assays to confirm that the observed antimicrobial effects were not influenced by the base matrix itself.

### 4.2. Preparation of Nigella sativa (NS) Formulation

An inert, commercial, water-soluble hydrogel (KY Jelly, Reckitt Benckiser, Slough, UK) was utilized as the vehicle base due to its non-irritating and optimal diffusion properties. To formulate the experimental antimicrobial agent, pure, cold-pressed NS seed oil was geometrically incorporated into the hydrogel base to achieve a final concentration of 5% (*w*/*w*). This specific 5% (*w*/*w*) concentration was selected based on preliminary pilot screening, representing the maximum stable oil-loading capacity achievable within this inert hydrogel vehicle without inducing phase separation or requiring the addition of synthetic emulsifiers. The mixture was continuously triturated under aseptic conditions until a homogenous, stable, and visually uniform emulsion was achieved. To prevent oxidation and degradation of the bioactive quinone constituents, the final NS formulation was protected from ambient light in opaque containers and stored at 4 °C. The formulation was freshly prepared prior to each experimental replicate to ensure maximum stability and bioavailability of the active compounds.

### 4.3. Agar Well Diffusion Model (In Vitro)

Initial antimicrobial potency and drug release kinetics were evaluated via a modified well diffusion protocol. Because standard CLSI broth microdilution (MIC) assays are highly unreliable for evaluating the intrinsic potency of viscous, hydrophobic emulsions due to phase separation in aqueous media, this diffusion assay was specifically utilized to validate the successful release of the bioactives from the hydrogel vehicle. Following spectrophotometric (Thermo Fisher Scientific, Waltham, MA, USA) calibration to a 0.5 McFarland standard (∼10^8^ CFU/mL), bacterial suspensions were uniformly swabbed onto Mueller Hinton agar containing a standard 1.7% (*w*/*v*) agar concentration to ensure consistent diffusion kinetics to establish a continuous growth lawn. Uniform 6 mm cavities were created using a sterile borer (VWR International, Radnor, PA, USA), and approximately 130 to 150 mg of the respective test agents (NS gel, standard positive controls, or vehicle) were loaded into each cavity. The plates were wrapped tightly to maintain a humidified environment and incubated for 24 h at 37 °C. Efficacy was determined by utilizing digital calipers (Mitutoyo Corporation, Kawasaki, Japan) to record the absolute diameter of the resulting clear zones.

### 4.4. Gauze Contact Biofilm Model (In Vitro)

A modified contact assay was employed to model the occlusive conditions of a topically treated wound bed. To initiate acute colonization, 10 µL of bacterial inoculum (10^2^–10^3^ CFU) was seeded onto sterile 6 mm cellulose matrices resting on an LB agar base. Standard 4 cm^2^ (2 cm × 2 cm) cotton gauze segments were saturated with 400 mg of the evaluated formulations and placed directly over the seeded matrices. Small, standardized weights were applied to guarantee flush physical contact. After a 24 h incubation at 37 °C, the underlying cellulose matrices were extracted, submerged in phosphate-buffered saline, and subjected to vigorous vortexing to dislodge the biofilm. Bacterial survival was subsequently quantified through serial dilution and colony enumeration. The theoretical limit of detection (LOD) for this assay was 10 CFU/disk (based on plating 100 µL from a 1 mL recovery suspension); “no growth” was defined as the complete absence of visible colonies at this lowest dilution, indicating a bacterial burden below the detection threshold.

### 4.5. Porcine Ear Skin Model (Ex Vivo)

Tissue penetration and efficacy against epidermal lipid barriers were investigated using porcine explants as a human skin analogue. Freshly acquired porcine ears underwent surface decontamination via 70% ethanol, followed by the surgical removal of underlying adipose layers to isolate the intact epidermis. The resulting tissue was cut into uniform 4 cm^2^ squares, and each segment received a 50 µL microbial challenge (10^7^ CFU/mL). Explants were transferred to a humidified environment for 1 to 2 h to facilitate bacterial attachment. Subsequently, a continuous coating of the test formulations was applied to the inoculated surfaces. To quantify time-kill kinetics, bacterial recovery was performed by vigorously swabbing the epidermal surfaces at 6 and 24 h post-treatment. The collected samples were then suspended in PBS, diluted, and plated for CFU analysis.

### 4.6. Galleria mellonella Topical Model (In Vivo)

The in vivo trans-cuticular efficacy and systemic protective capacity of the NS formulation were evaluated using *Galleria mellonella* larvae, serving as a macroscopic, ethical surrogate for vertebrate skin infection models. Healthy, final-instar larvae weighing 200–300 mg were procured, starved for 24 h to normalize baseline metabolic activity, and maintained in darkness at 15 °C prior to use. Larvae were injected via the dorsal cuticle with 10 µL of the bacterial suspension (10^3^–10^4^ CFU) to establish a lethal systemic infection. Immediately following the pathogenic challenge, the NS formulation and control agents were applied directly to the dorsal cuticular surface using a sterile applicator. The treated larvae were incubated at 37 °C for 24 h. Post-incubation, surviving and deceased larvae were mechanically homogenized in sterile saline. The resulting larval homogenate was serially diluted and plated to quantify the systemic bacterial burden (CFU/larva).

### 4.7. Statistical Analysis

Quantitative outcomes are expressed as the mean ± standard deviation (SD). All experiments across all in vitro, ex vivo, and in vivo models were performed utilizing exactly four independent biological replicates (*n* = 4) per treatment group. While a formal a priori power analysis was not conducted, this sample size was established in accordance with standard methodological precedents for exploratory pre-clinical antimicrobial screening to adequately capture anticipated multi-log bioburden reductions. Prior to parametric testing, the assumptions of normality and homogeneity of variances were verified utilizing the Shapiro–Wilk and Levene’s tests, respectively, with non-normal bacterial recovery data undergoing log-transformation to satisfy these criteria. To determine variances between the experimental formulations and vehicle cohorts, we applied an independent two-tailed Student’s t-test or a one-way analysis of variance (ANOVA) coupled with a Tukey multiple comparison post hoc procedure, contingent on the data structure. To quantify the magnitude of the observed outcomes alongside null-hypothesis testing, 95% confidence intervals (CIs) were computed, and effect sizes were calculated utilizing Hedges’ *g* to appropriately correct for the small sample size. Statistical relevance was recognized when the probability value fell below 0.05, and findings were classified as highly significant at *p* < 0.001. All computational tasks and inferential testing were executed utilizing the SciPy library (version 1.11) within Python (version 3.10; Python Software Foundation, Wilmington, DE, USA).

### 4.8. Ethics Statement

All experimental methodologies were developed in strict accordance with the 3Rs ethical principles (Replacement, Reduction, Refinement). Because *Galleria mellonella* constitutes an invertebrate species, institutional animal care and use committee (IACUC) oversight is not mandated by contemporary regulatory standards. Similarly, the porcine tissue evaluated in the ex vivo assays was reclaimed entirely from commercial slaughterhouse waste streams, explicitly exempting this research from requiring formal vertebrate ethical review.

## 5. Conclusions

The novel 5% NS seed oil gel represents a potent, broad-spectrum, plant-based pre-clinical candidate with the potential to serve as an adjunctive or alternative agent to standard topical antibiotics like mupirocin. Validated across a robust, multi-tiered preclinical platform—specifically utilizing highly translational porcine skin and *Galleria mellonella* models—the formulation demonstrated exceptional efficacy in significantly reducing resilient Gram-positive pathogens, particularly within localized, lipid-rich wound environments. In addition, it exhibited activity against *P. aeruginosa*. These findings strongly position the NS formulation as a highly promising pre-clinical candidate for the decolonization and management of complex, polymicrobial wound infections. By successfully navigating this tiered pre-clinical platform, the formulation establishes a robust foundation justifying future in vivo mammalian wound healing models, which remain a strict prerequisite for definitive clinical translation.

## Data Availability

The original contributions presented in this study are included in the article. Further inquiries can be directed to the corresponding author.
